# Volatile Organic Compounds Gas Sensors Based on Molybdenum Oxides: A Mini Review

**DOI:** 10.3389/fchem.2020.00339

**Published:** 2020-05-05

**Authors:** Jingxuan Wang, Qu Zhou, Shudi Peng, Lingna Xu, Wen Zeng

**Affiliations:** ^1^College of Engineering and Technology, Southwest University, Chongqing, China; ^2^Chongqing Electric Power Research Institute, State Grid Chongqing Electric Power Company, Chongqing, China; ^3^College of Materials Science and Engineering, Chongqing University, Chongqing, China

**Keywords:** MoO_3_, gas sensors, volatile organic compounds, functional modification, gas-sensing mechanism

## Abstract

As a typical n-type semiconductor, MoO_3_ has been widely applied in the gas-detection field due to its competitive physicochemical properties and ecofriendly characteristics. Volatile organic compounds (VOCs) are harmful to the atmospheric environment and human life, so it is necessary to quickly identify the presence of VOCs in the air. This review briefly introduced the application progress of an MoO_3_-based sensor in VOCs detection. We mainly emphasized the optimization strategies of a high performance MoO_3_, which consists of morphology-controlled synthesis and electronic properties functional modification. Besides the general synthesis methods, its gas-sensing properties and mechanism were briefly discussed. In conclusion, the application status of MoO_3_ in gas-sensing and the challenges still to be solved were summarized.

## Introduction

Volatile organic compounds (VOCs) mainly come from the exhaust gases generated by fuel combustion and transportation, as well as emissions from building materials, decorative materials, and furniture. People can suffer headaches, nausea, and even more severe issues such as convulsions and comas when exposed to a certain concentration of VOCs (Chu et al., 2010; Sui et al., 2015). Moreover, many carcinogens that damage the liver, kidneys, brain, and nervous system were found in VOCs. Therefore, the problem of air pollution by VOCs has attracted extensive attention in many countries (Yang et al., 2018; He S. H. et al., 2019). Currently, there are two common techniques—photo ionization detector (PID) and flame ionization detector (FID)—to detect VOCs, however, the application of these methods in industry are limited due to the relatively high cost and complicated maintenance. Considering the characteristics of small size, low cost, and convenient fabrication, semiconductor gas sensor technology plays an important role in many fields (Lu et al., 2018; Xiao et al., 2018; Zhang D. Z. et al., 2018; Zhang Q. Y. et al., 2018; Zhou et al., 2018c, 2019; Wang et al., 2019a; Wei et al., 2020), so it is reasonable to propose the employment of a gas sensor to realize the online monitoring of VOCs.

As a typical n-type semiconductor material with a suitable band gap (2.39–2.9 eV) (Yan et al., 2016), MoO_3_ has attracted wide attention because of its distinctive gas sensing performances in the detection of many gases (Liu et al., 2015; Xia et al., 2016; Li, 2017; Zhou et al., 2017; Yang et al., 2018). Researchers have been devoted to designing nanomaterials with more suitable properties, and two methods have proved effective through unremitting efforts. One is to synthesize materials with larger specific surface areas, which is attributed to the conclusion that the micromorphology features have an impact on the gas-sensitive process (Zhou et al., 2018b; Zhu et al., 2018; Wang et al., 2019b; Wei Z. J. et al., 2019). Considering the changes in the materials that are caused by doping and compounding or the catalytic effect of the introduced material on the sensing process, it is also desirable to improve the properties of the materials by introducing other elements or substances (Mousavi-Zadeh and Rahmani, 2018; Zhou et al., 2018a; Wang et al., 2019c; Xu et al., 2019). Therefore, the optimization strategies of MoO_3_ based on controllable morphology synthesis and functional modification were comprehensively summarized in this mini review. Besides, several typical synthesis pathways of MoO_3_ nanomaterials, as well as the gas-sensing performances and mechanism to VOCs, were introduced.

## Synthesis Methods of MOO_3_

The preparation of materials with more useful properties has always been a research hotspot in the field of gas detection, and has attracted extensive attention from scholars. In recent years, material preparation craft is constantly updated and developed with the emergence of new technology. At present, solid phase method, liquid phase method, and template method are mainly employed to synthesize MoO_3_ materials with admirable performance.

The process of preparing materials by solid phase method is to transform the solid phase raw materials into target powders. Using ammonium molybdate as raw material, Qin et al. (2017) successfully obtained MoO_3_ nanoplate arrays in the air through a solid phase chemical synthesis route. By hydrolyzing a mixture of one or more soluble metal salts solution and then evaporating and sublimating them, the liquid phase method adopts a series of processes to separate the solute from the solvent, where finally the nanoparticles with uniform shape are produced by crystallizing metal ions. Nowadays, spray pyrolysis technique, sol-gel route, and hydrothermal method have been reported as common liquid phase methods for the preparation of MoO_3_ materials. Sau et al. (2019) used the sol-gel method to heat the solution after the molybdenum source was fully dissolved to a gelatinous state under specific PH (7–8) conditions. Finally they prepared α-MoO_3_ nanoparticles through annealing and drying. Pandeeswari and Jeyaprakash (2014) successfully obtained MoO_3_ thin films with a thickness of 520 nm on a glass substrate maintained at 250°C by spray pyrolysis route. Zhu et al. (2019) synthesized hollow MoO_3_ microcages by a facile one-step hydrothermal process, which had gone through four steps of heating, cooling, centrifugation, and washing. The template method is designed to generate nanomaterials based on the template of appropriate structure, which can effectively influence the growth direction and morphology of the materials. Zhang et al. (2017) deposited MoS_2_@MnCO_3_ powder based on an MnCO_3_ template, and prepared MoS_2_ by adding hydrochloric acid to remove MnCO_3_. Finally, they obtained hierarchical MoO_3_ microboxes by calcining MoS_2_ powder.

## Optimization Strategies OF MOO_3_

The sensitivity of the gas sensors is closely related to the changes in the resistance that is attributed to the adsorption and desorption of target gas molecules on the surface of materials, which implies that the gas-sensing properties mainly depend on their own electronic characteristics (carrier concentration, energy band structure, etc.) and morphological characteristics (specific surface area, aperture, etc.). Based on this, the main strategies to enhance the gas-sensing performances of materials are morphology control and electronic properties improvement.

### Controllable Synthesis of Diversified MoO_3_

**Figure 1:**
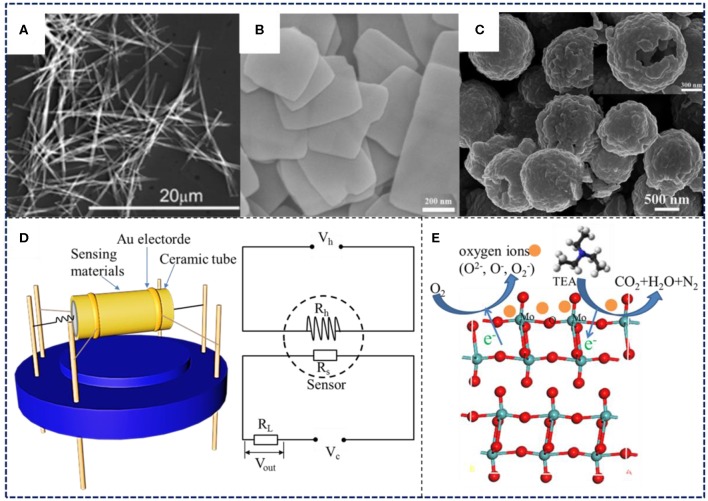
**(A)** Nanoribbons. Reprinted with permission from Kwak et al. Copyright (2019) American Chemical Society. **(B)** Nanosheets. Reprinted with permission from Shen et al. Copyright (2019) American Chemical Society. **(C)** Hollow microspheres. Reprinted with permission from Zhang et al. Copyright (2019) American Chemical Society. **(D)** Schematic diagram of sensor and detection circuit. **(E)** Gas sensing mechanism.

In recent years, the production of MoO_3_ gas-sensitive materials with high quality morphology has become an important research approach for performance enhancement. In this regard, MoO_3_ gas-sensitive materials with different morphologies and dimensions have been prepared through different methods to conduct gas-sensing experiments. In this section, we review and summarize the excellent achievements of MoO_3_ morphology control and several typical morphologies are shown in Figures 1A–C.

One-dimensional (1D) structures, such as nanofibers, nanorods, and nanoribbons, have limited specific surface areas, but their reaction sites are directly exposed to external environments, leading to the susceptive variation of interface ion transport when some changes occur to environmental factors. Hence, 1D material has the potential to be applied as excellent sensors. The remarkable selectivity and response-recovery characteristics of α-MoO_3_ nanorods gas sensors to triethylamine was reported (He S. H. et al., 2019). Besides, the rapid adsorption/desorption processes were confirmed to be the reason for prominent gas-sensing characteristics. By optimizing reaction time and pulse temperature in hydrothermal reaction, Mandal et al. (2019) synthesized uniform MoO_3_ nanobelts and MoO_3_ nanofibers. The experimental results illustrated that the enhanced ethanol detection performances of nanofibers could be ascribed to the larger specific surface area and surface defects compared with the nanowires.

Materials with two-dimensional structures such as nanoplates and nanosheets have a larger specific surface area, which further improves the gas-sensitive responses. The prominent electron depletion layer that derived from the thin morphologic features of nanoplates was verified as beneficial due to the ultrahigh gas-sensing performances. Moreover, the porous structure caused by the combination of nanoplates could effectively facilitate gas diffusion on the surface of MoO_3_, providing convenience for the gas-sensing process (Cho et al., 2014). The study of Jiang et al. (2018) demonstrated that MoO_3_ microsheets with a large number of oxygen vacancies exhibit superior sensing properties, and the strong reaction between adsorbed oxygen and target gases to be confirmed was also an important factor to promote the gas-sensing performances.

Three-dimensional structures with large specific surface areas are generally assembled from low-dimensional structures, which have more reaction sites for gas adsorption, leading to better gas-sensitive responses and lower gas detection limits. Furthermore, more gas diffusion channels are provided by the assembled porous structure, which are conducive to gas sensitivity reaction. Common three-dimensional structures of MoO_3_ include nanoarrays, nanoflowers, and nanospheres. For example, Ji et al. (2019a) synthesized three hierarchical MoO_3_ flower-like samples with different petal shapes and found that the material with the thinnest petals had the most outstanding gas-sensing performance. Specifically, the thin nanosheets assembled flower-like MoO_3_ has a larger specific surface area that provides more potential for gas-sensitive reactions, and the thinness allows electrons to transfer rapidly across the surface, which implied the procedure of gas-sensing became easier. In addition, the curved edges of thin petals make it harder for gas molecules to leave the surface, facilitating the full process of adsorption. Cong et al. (2016) proved the sensors based on MoO_3_ nanoarrays assembled from a large number of long nanorods exhibited more reactive sites and more active surface electrical properties than the nanoarrays with fewer nanorods.

### Functional Modification Methods

Functional modification is of great significance to improving the properties of gas-sensitive materials. In the present studies, the modification of MoO_3_ mainly includes two approaches of element doping and multi-component compounding, which have been proven to be effective methods in optimizing the electronic properties of materials. In this section, the advances of MoO_3_ modification research were briefly summarized.

Metal doping is an alternative approach to acquire the modified characteristics of MoO_3_. On the one hand, suitable metal doping can effectively reduce the activation energy of chemisorption reaction for the measured gases. On the other hand, the metal elements play the role of the catalytic activity center, leading to the optimization of the gas-sensing performances. Scholars have studied the influence of different doping elements on the properties of MoO_3_ materials. For instance, Cr-doped MoO_3_ nanorods had more oxygen vacancy induced by doping, which is meaningful for the promotion of sensitivity (Li et al., 2019). Similar results were obtained when the W element was doped in MoO_3_ nanobelts (Li et al., 2017b). As for the dope of Ni, not only did more adsorbed oxygen that can promote the change of resistance in the sensing process appear on the surface of MoO_3_, but also the morphology changed with the increase of the doping amount, which is related to the inhibition of lattice growth by the introduction of doped Ni elements (Jiang et al., 2019). Yang et al. (2017) prepared Zn-doped MoO_3_ nanobelts using the hydrothermal method. They found that the doped zinc caused the reduction of the band gap of MoO_3_ and increased the amount of adsorbed ethanol molecules, which contributed to the excellent performance.

Many researchers have been devoted to the preparation of hybrid structures that affected properties such as grain boundary barrier, energy band, carrier concentration, and depletion layer, thus improving the performance of gas sensing. For instance, the Au nanoparticle with a larger work function than MoO_3_ received electrons from MoO_3_ nanosheets, leading to the appearance of electron depletion layer at the Au/MoO_3_ Schottky junction, and the enhanced ethanol detection capabilities were attributed to the resulting high resistance (Yan et al., 2016). Considering Pt nanoparticles combined MoO_3_ nanobelts, the superior selectivity to formaldehyde was conducted, which was ascribed to the catalytic effect of loaded particles on formaldehyde during the surface gas sensing process (Fu et al., 2019). As for the RuO_2_ nanoparticles modified MoO_3_ nanobelts, oxygen vacancies produced on the surface, creating more adsorption-desorption sites for gas molecules (Wei Q. et al., 2019). With regard to heterostructure, Li et al. (2018) synthesized the CoMoO_4_/MoO_3_ nanostructure with p-n heterojunction using the dipping-annealing method. The enhancement of the adsorption to oxygen by p-type CoMoO_4_ and the barrier formed at the p-n junction were verified to be favorable to the improved gas response.

Above all, previous studies have focused on the optimization of MoO_3_ through synthesizing different samples with multiple morphologies, the doping of transition metals such as Cr, W, Ag, Au, Fe, Zn, and Ni, and decorating with other nanomaterials (Au nanoparticles, RuO_2_ nanoparticles, Fe_2_O_3_ nanoparticles, CoMoO_4_ nanoparticles, NiCo_2_O_4_ nanosheets, etc.). Thus, in order to improve the gas sensitivity of MoO_3_, many potential materials for the modification of innovative synthesis methods with controllable morphology need to be explored.

## Gas-Sensing Application of MOO_3_

### Gas-sensing Mechanism of MoO_3_

The theory of sensitivity generated by changes in material resistance during the gas-sensing process has been widely accepted by scholars in the investigation of the gas-sensitive mechanism of metal oxides. As shown in Figure 1E, the reaction between the target gas molecules and the adsorbed oxygen ions (${\text{O}}_{2}^{-}$, O^−^, O^2−^) on the surface of the gas-sensitive materials leads to the change of the electrical conductivity, which is key to detecting the corresponding response (Li et al., 2015). MoO_3_ is an n-type semiconductor with electrons as internal carriers. Mass of oxygen molecules in the air tends to be adsorbed by the MoO_3_, forming adsorbed oxygen ions accompanied by the acceptance of electrons from the conduction band of MoO_3_. Thus, the electron depletion layer was formed on the surface of MoO_3_, which caused the increase of resistance (Ji et al., 2019a). The specific process can be expressed by the following equation:

(1) $$\begin{array}{l} O_{2\left(gas\right)}\to O_{2\left(ads\right)} \end{array}$$

(2) $$\begin{array}{l} O_{2\left(ads\right)}+e^{-}\to {{O_{2}}^{-}}_{\left(ads\right)} \end{array}$$

(3) $$\begin{array}{l} O_{2\left(ads\right)}+{2e}^{-}\to {2O^{-}}_{\left(ads\right)} \end{array}$$

(4) $$\begin{array}{l} {O^{-}}_{\left(ads\right)}+e^{-}\to {O^{2-}}_{\left(ads\right)} \end{array}$$

When the MoO_3_ sensor was exposed to the atmosphere of the target gas, the adsorbed oxygen ions underwent a reduction reaction. Further, electrons are released back into the conduction band of MoO_3_, and the depletion layer narrows, which results in a decrease in the resistance of the material and completes the whole gas sensing process. Take triethylamine for example, this process can be expressed as the following equation (Wei Q. et al., 2019).

(5) $$\begin{array}{l} {{2\left(C_{2}H_{5}\right)}_{3}N}_{\left(gas\right)}+39{O^{-}}_{\left(ads\right)}\to N_{2\left(gas\right)}+{12CO}_{2\left(gas\right)}\\ \;+15{H_{2}O}_{\left(gas\right)}+39e^{-} \end{array}$$

### Gas Sensing Properties of MoO_3_

Nowadays, many researchers focus on the application of MoO_3_ materials in gas sensors, while the ultimate goal of the investigation is to obtain higher performance MoO_3_-based sensing materials. Notably, MoO_3_ with prominent gas-sensing properties has been proven to be an alternative sensing material to detect VOCs. We summarized the representative research on VOCs detection, which mainly focused on the use of formaldehyde (HCHO), methanol (CH_3_OH), ethanol (CH_3_CH_2_OH), xylene (CH_3_C_6_H_4_CH_3_), trimethylamine ((CH_3_)_3_N), and triethylamine ((C_2_H_5_)_3_N), and listed them in Table 1. Figure 1D shows the structure diagram of the side heat sensor and the gas-sensing test circuit. The sensing-materials were coated on the alumina ceramic tube. A Ni-Cr resistance wire that could conveniently control the current was inserted in the coating tube and the change of resistances was tested by electrodes. The gas response (S) of MoO_3_ based sensors to reducing gas is calculated by S = *R*_*a*_/*R*_*g*_, while to oxidizing gas it is S = *R*_*g*_/*R*_*a*_ (Jiang et al., 2019).

**Table 1:** Summary of recent researches on MoO_3_-based sensors for VOCs detection.

**Gas**	**Sensing material**	**Concentration(ppm)**	**Temperature (^**°**^C)**	**Response**	**References**
Formaldehyde	Ni-doped -MoO_3_ nanolamella	100	255	41	Shen et al., 2017
	Pt-decorated MoO_3_ nanobelts	200	27	19.1%	Fu et al., 2019
Methanol	α-MoO_3_ nanorod arrays	500	300	7.8	Cong et al., 2016
	ZnO microcube/MoO_3_ micrograss	500	200	56	Mandal et al., 2018
Ethanol	nanofiber-assembled hierarchical MoO_3_	400	300	32	Ji et al., 2019b
	Au nanoparticles/MoO_3_ nanobelts	500	200	50	Wang et al., 2018
	α-MoO_3_ nanobelts	500	300	80	Mo et al., 2020
	Zn-doped MoO_3_ nanobelts	250	240	52	Yang et al., 2017
Xylene	α-MoO_3_ nanoarrays	1,000	370	83.9	Qin et al., 2017
	Fe-doped α-MoO_3_ nanoarrays	1,000	340	166.3	Wang et al., 2020
	Fe_2_O_3_ nanoparticles/MoO_3_ nanobelts	100	233.5	22.48	Qu et al., 2019
	Ni-doped MoO_3_ nano-pompon	100	250	62.6	Jiang et al., 2019
Trimethylamine	Ce-doped MoO_3_ nanobelts	50	240	17.4	Li et al., 2017a
	Au nanoparticles/MoO_3_ nanobelts	50	280	70	Zhang et al., 2016
	porous α-MoO_3_ nanosheets	10	133	51.47	Shen et al., 2019
	MoO_3_ nanobelts	50	240	582.3	Yang et al., 2016
Triethylamine	MoO_3_ microsheets	100	275	27.1	Jiang et al., 2018
	α−*Fe*_2_*O*_3_/α-MoO_3_ nanostructure	50	280	76	Liu et al., 2019
	Cr-doped α-MoO_3_ nanorods	100	200	150.25	Li et al., 2019
	Ag nanoparticles/α-MoO_3_ nanorods	100	200	408.6	He K. et al., 2019

## Conclusion

This mini review focused on the latest advances in synthetic methods, morphological control, functional modification, and gas-sensing application including properties and mechanism of MoO_3_ materials in the detection of VOCs. The studies of morphologically-controlled synthesis proved that MoO_3_ with a high specific surface area possesses superior gas-sensing performances and provided reference experience for further MoO_3_ gas-sensing material design. Further, appropriate element doping or material hybridization could improve properties of MoO_3_, such as energy band gap and adsorbed oxygen content, which is advantageous to the gas sensing process. Scholars have made great efforts to develop more efficient MoO_3_-based VOCs sensors and have shown objective achievement, but there are still challenges in practical application. The design of porous structures or hierarchical structures with more reactive gas pathways and reaction sites to further improve the specific surface area of MoO_3_ material is an issue that needs to be further explored. In addition, more alternative modification materials should be selected through experimental verification. All of these issues should continue to be addressed to obtain MoO_3_-based materials with a higher response, better selectivity, superior stability, and lower operating temperature. Finally, the gas-sensing mechanism was not complete. By combining theoretical calculation and analysis, the changes of electronic properties at the micro level should be analyzed, which will allow for further understanding of the nature of gas sensing and provide guidance for designing MoO_3_ materials with better gas-sensing performances in the future.

## Author Contributions

All authors listed have made a substantial, direct and intellectual contribution to the work, and approved it for publication.

## Conflict of Interest

The authors declare that the research was conducted in the absence of any commercial or financial relationships that could be construed as a potential conflict of interest.
